# Chernobyl nuclear catastrophe: lessons for sustainability and UNSDGs in health, energy, and environmental recovery

**DOI:** 10.3389/fpubh.2025.1552122

**Published:** 2025-02-26

**Authors:** Chee Kong Yap, Khalid Awadh Al-Mutairi

**Affiliations:** ^1^Department of Biology, Faculty of Science, Universiti Putra Malaysia, Selangor, Malaysia; ^2^Department of Biology, Faculty of Science, University of Tabuk, Tabuk, Saudi Arabia

**Keywords:** Chernobyl, nuclear contamination, radiation health, environmental implications, sustainability

## Abstract

This study provides a comprehensive review of the research surrounding the Chernobyl nuclear incident, focusing on its far-reaching impacts on human health, and environmental contamination. Based on the Scopus database, 258 relevant papers were identified using Preferred Reporting Items for Systematic Reviews and Meta-Analyses (PRISMA) guidelines. These papers were metal-analyzed and quantitatively analyzed using a similarity map generated through VOSViewer in order to visualize key themes and their interconnections. The research highlights critical areas such as radiation-induced health effects, ecological damage, and the implications for sustainable energy practices. Additionally, this review explores the alignment of these findings with several United Nations Sustainable Development Goals (UNSDGs), particularly UNSDG 3 (Good Health and Wellbeing), UNSDG 6 (Clean Water and Sanitation), UNSDG 7 (Affordable and Clean Energy), UNSDG 13 (Climate Action), and UNSDG 15 (Life on Land). By synthesizing existing research, this study emphasizes the importance of integrating safety protocols, environmental rehabilitation, and sustainable energy policies to prevent and to mitigate the impacts of future nuclear incidents.

## 1 Introduction

The year 1986 is marked by two significant global events: the Mexico FIFA World Cup and the Chernobyl Nuclear Disaster (CND). The analysis of public attention toward these events prompts an inquiry into which incident achieved greater global awareness and impact. From a socio-environmental perspective, the 1986 CND was prominently reported in local and international newspapers, as well as in reports from the International Atomic Energy Agency (IAEA), including forums and conferences, and in academic literature. Numerous reports indicate that the CND is regarded as one of the most catastrophic technological and environmental incidents in human history. This analysis considers the persistent significant impacts on human health, ecological redistribution, and global energy policies (1–4). The failure of Reactor No. 4 at the Chernobyl Nuclear Power Plant released significant amounts of radioactive materials into the atmosphere. This has led to significant contamination across various European nations, including Ukraine, Russia, and Belarus (5–7). The immediate health effects, including acute radiation syndrome and the mandatory evacuation of over 300,000 people, initiated a prolonged global response to this unprecedented nuclear disaster (8–10). Recent studies demonstrate the lasting impacts of the catastrophe on human health, the environment, and energy security, underscoring its significance in comprehending the risks associated with nuclear power (11–13).

The CND provides important insights relevant to the objectives set by the United Nations Sustainable Development Goals (UNSDGs) (14). Nuclear power is a contentious option in the global transition to clean energy for mitigating climate change, largely because of the potential for catastrophic events, as illustrated by CND (15). Secure, low-carbon energy sources are crucial; however, the CND disaster underlines the importance of stringent safety standards and preparedness for emergencies within the energy sector (16). The CND stresses the necessity of balancing energy efficiency with environmental safety in climate change initiatives (17). The health effects of Chernobyl have been the subject of extensive research. The CND resulted in a rise in cases of radiation-54-related malignancies, thyroid disorders, psychological distress, and possible genetic anomalies, thereby raising substantial public health concerns (18).

The CND had a disproportionate impact on children and pregnant women, resulting in long-term psychological trauma for survivors, which requires continued epidemiological research and healthcare support (18–20). The environmental consequences were significant, affecting soil, water, and ecosystems, with detrimental impacts on biodiversity and food security, necessitating restoration and mitigation efforts (21). The exclusion zone has emerged as an ecological research site, illustrating nature’s resilience in the face of ongoing contamination (22). The disaster underscored the necessity for institutional and policy reforms, highlighting the significance of transparent governance, crisis management, and international cooperation in mitigating nuclear risks (23, 24). The Soviet government’s inability to deliver timely information and effectively manage the crisis exacerbated its consequences, highlighting the importance of robust institutions, international dialogue, and revised nuclear safety regulations to avert future disasters (25, 26).

Extensive research over the decades has examined the environmental, health, and remediation aspects of the CND. The IAEA (27) conducted an initial evaluation via The International Chernobyl Project, detailing both the immediate and long-term consequences of the accident. Ten years later, a detailed summary of the outcomes was recorded, highlighting the socio-economic and health effects of radiation exposure (28). Subsequent analysis examined the environmental consequences of the disaster, outlining remediation strategies and insights gained over two decades (29). IAEA (30) highlighted the significance of historical lessons in informing future nuclear safety policies. In 2019, the IAEA evaluated the environmental effects of the cooling pond drawdown at the Chernobyl Nuclear Power Plant, aiding in the ongoing decommissioning and ecological recovery efforts. These studies collectively underscore the importance of ongoing monitoring, remediation, and policy development to address the long-term effects of CND.

This review acknowledges the incorporation of references from IAEA reports regarding the CND. The current study is based on a thorough review of peer-reviewed literature indexed in Scopus, which ensures rigorous academic scrutiny and broad scientific consensus. The incorporation of IAEA reports, which mainly reflect institutional viewpoints, may result in a bias that contrasts with independent research outcomes recorded in Scopus database studies. The Scopus database includes numerous independent research articles that analyze the long-term health, environmental, and socio-economic impacts of the CND, facilitating a diverse and nuanced discussion. IAEA reports serve as important official documentation; however, their institutional perspective frequently prioritizes regulatory and policy interpretations over independent empirical research. This review paper includes a brief mention of significant IAEA reports to acknowledge their perspective, while prioritizing peer-reviewed empirical research sourced from the Scopus database to maintain academic integrity.

This review paper aims to assess insights gained from the CND and evaluate their significance in relation to the UNSDGs, utilizing the Scopus database. This review aims to clarify the important connections between nuclear safety, environmental resilience, and sustainable development by analyzing the long-term impacts of the CND on public health, environmental management, and global governance.

## 2 Methodology

This research utilized a bibliometric analysis to examine the scientific literature concerning the CND. Bibliometric analysis serves as a quantitative approach for evaluating the influence and development of scientific research through the examination of publication and citation trends (31). This methodology sought to delineate the scope and principal trends in research pertaining to Chernobyl, as recorded in the Scopus database during the period from 1986 to 2024.

**Figure 1:**
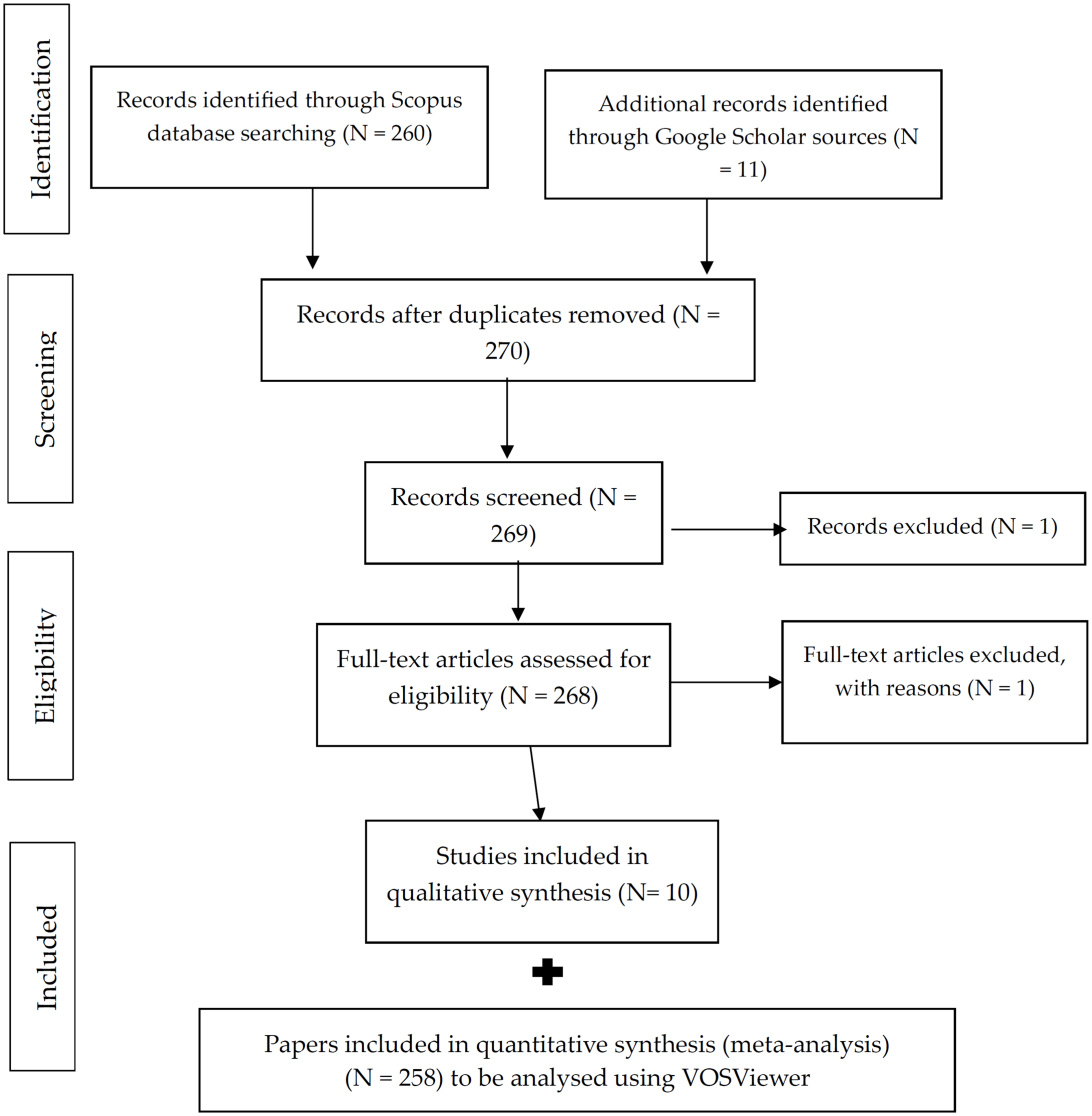
Flowchart of the Preferred Reporting Items for Systematic Reviews and Meta-Analyses (PRISMA) [adapted from Moher et al. (32)], used in the present study.

This review utilized the Systematic Literature Review methodology in accordance with the Preferred Reporting Items for Systematic Reviews and Meta-Analyses (PRISMA) guidelines established by Moher et al. (32) to enhance understanding of the “Chernobyl Disaster.” PRISMA offers a framework grounded in evidence to promote transparency and facilitate critical evaluation in research. Figure 1 illustrates the formal steps adapted for this study. Scopus was selected as the primary database because of its extensive and multidisciplinary coverage of high-quality, peer-reviewed content, establishing it as a reliable resource for academic research (33). A keyword search for “Chernobyl Disaster” was performed in Scopus, encompassing publications from 1986 to October 10, 2024. Only abstracts containing relevant keywords and addressing significant issues were included. The initial selection aimed to minimize bias by focussing exclusively on paper titles that included the specified keywords, without considering authors’ names or countries of origin. This approach facilitated an objective and systematic selection of literature for analysis.

A total of 258 papers have been included for quantitative synthesis (meta-analysis) and will be analyzed using VOSViewer (version 1.6.20; 2009–2023 Van Eck & Waltman; Leiden University, The Netherlands). The metadata was imported into VOSviewer, a software specifically developed for constructing and visualizing bibliometric networks (31). VOSviewer was employed to manage extensive datasets and illustrate intricate relationships among terms in the literature. The software constructs a network utilizing the frequency of term co-occurrence in the chosen publications. Co-occurring terms were often linked by edges, with the strength of these connections indicating the level of co-occurrence (34).

VOSviewer subsequently employed its clustering algorithm to categorize related terms into distinct clusters. Each cluster represented a distinct research theme or topic, with clusters color-coded for enhanced interpretation. This visualization method facilitated a clear comprehension of the primary research domains and their relationships (35). The identified clusters revealed important research topics and their interconnections, emphasizing emerging trends in the investigation of the CND (31, 34, 36).

The clustering analysis facilitates the mapping of research themes related to the UNSDGs, specifically in the areas of health, environmental sustainability, and energy policy. The mapping of thematic clusters highlighted the significance of Chernobyl-related research in relation to global sustainability and planetary health issues.

## 3 Results and discussion

**Figure 2:**
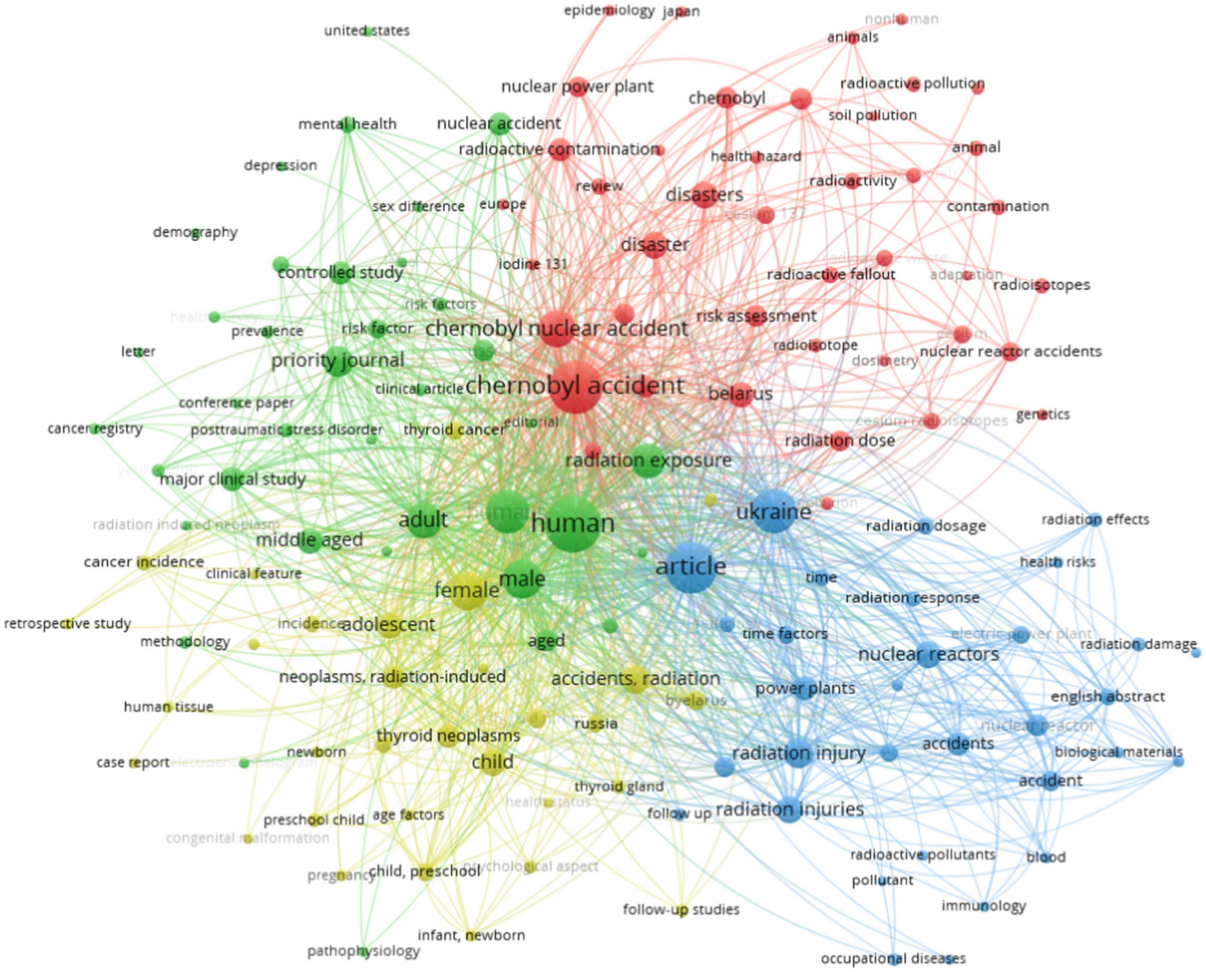
Visualization of similarity using VOSviewer based on 258 publications indexed in the Scopus database from 1986 to 2024 using the keyword “Chernobyl Disaster” searched on 10 October 2024.

The Scopus database found 258 papers. This study’s results (Figure 2) illustrate the co-occurrence and interconnections among academic keywords related to the Chernobyl Disaster. Each node represents a keyword or concept, and the links between nodes indicate the strength of their association based on co-occurrence in academic literature.

The network visualization of the CND reveals four distinct clusters representing the main thematic areas of study related to this disaster (Figure 2). The clusters, identified by color, provide insights into the breadth of research topics and their interconnections. Each cluster is characterized by its own major keywords that shed light on the various facets of the CND and its aftermath.

### 3.1 Clustering interpretations

The network visualization of research related to the CND reveals four primary areas of focus, each characterized by a unique emphasis. This analysis will examine the implications of the research findings for each cluster and investigate the interrelationships among them.

#### 3.1.1 Red cluster: emphasis on environmental and geopolitical issues

The red cluster, focused on radioactive pollution and nuclear accidents, underscores the significant environmental and geopolitical consequences of the CND (37–39). Research on nuclear power plants and radioactive fallout analyses the immediate and long-term environmental impacts of disasters, particularly regarding soil, water, and air pollution (40–42). The distribution of radioisotopes such as iodine-131, cesium-137, and strontium-90 is crucial for evaluating the extent of environmental degradation (43, 44). The ongoing presence of these radionuclides in the environment highlights the seriousness of contamination, particularly in agricultural regions, where radioactive soil pollution has direct implications for food safety and human health (45, 46).

The geopolitical aspect of this cluster is underscored by mentions of Belarus, Ukraine, and nuclear reactor incidents, reflecting the regional focus of the majority of the research (47, 48). Ukraine and Belarus, the two nations most affected by the aftermath, have been the subject of numerous studies analyzing the accident’s social, political, and economic consequences (49, 50). Research indicates that nuclear disasters like Chernobyl place significant demands on governments, involving both immediate crisis response and long-term recovery efforts (51, 52). Radiation exposure levels in these nations pose a significant concern, impacting public health as well as agricultural and economic activities, given that extensive areas have remained polluted and uninhabitable for decades (53). The focus on radioactive and soil pollution underscores the ongoing challenges in mitigating environmental damage, indicating the need for ongoing monitoring and remediation efforts (54–56).

The findings have significant implications for international nuclear energy policy, influencing discussions regarding the safety of nuclear power as a viable energy source in the context of climate change mitigation (37). CND serves as a case study for evaluating global nuclear reactor readiness, emphasizing the critical needs for safety protocols, emergency responses, and international cooperation in the event of future nuclear incidents (20, 38).

#### 3.1.2 Green cluster: human health and epidemiology

The green cluster examines the human health effects of the Chernobyl accident, with an emphasis on epidemiological analysis (39, 57). Demographic effects of radiation exposure have been extensively studied, particularly concerning vulnerable groups such as females, males, children, adolescents, and infants (47, 48). Thyroid cancer, radiation-induced neoplasms, and post-traumatic stress disorder illustrate the significant medical and psychological effects on populations exposed to radiation. Thyroid cancer is notably one of the most well-documented health outcomes, especially in children and adolescents exposed to iodine-131 fallout (49, 50).

Research on cancer incidence and risk factors has shown a significant rise in radiation-induced malignancies, impacting thyroid tissues as well as various other organs (51, 52). Research indicates that children and adolescents exhibit heightened susceptibility to thyroid neoplasms due to the accumulation of radioactive iodine in the thyroid gland, primarily from contaminated milk and other dietary sources (53, 56). The terms prevalence and clinical aspects suggest that the study primarily aims to clarify the unique features of these malignancies, their latency periods, and the probability of recovery with early detection (55).

The psychological impact of the disaster has been considerable, as evidenced by the focus on mental health, and depression (54). Survivors, particularly those who have experienced displacement or high radiation exposure, exhibit persistent mental health challenges (20, 37). This research emphasizes the need for comprehensive mental health services for Chernobyl survivors, many of whom continue to face trauma related to displacement, loss, and ongoing health issues (38, 41). This cluster emphasizes the demographics and health conditions of communities exposed to radiation, suggesting that comprehensive epidemiological investigations are crucial for understanding the broader public health implications of nuclear disasters (40, 42).

This cluster presents concerning implications for reproductive health, as indicated by terms such as pregnancy, congenital abnormalities, and infants (43, 44). Research in this field has shown that radiation exposure can lead to increased rates of congenital anomalies and developmental impairments, affecting future generations in regions exposed to radiation fallout (46). The findings highlight the intergenerational health effects of the Chernobyl disaster, underscoring the need for extended health monitoring and support for the impacted communities (45).

#### 3.1.3 Blue cluster: radiological harm and technical investigations

The blue cluster presents a technical perspective, emphasizing radiation injuries, nuclear reactors, and radiation dosage (47, 48). This research aims to clarify the mechanisms of radiation damage and to develop criteria for measuring radiation exposure in both acute and chronic contexts (49, 50). Terms like radiation dose, radiation response, and radiation damage suggest that a considerable focus of this research is on dosimetry, which involves the quantification and assessment of the radiation dose absorbed by individuals, as well as the biological reactions to varying radiation levels (51, 52).

The terms nuclear reactors, radiation impacts, and accident pertain to research that investigates the technical aspects of nuclear power generation and the deficiencies that led to the Chernobyl disaster (53, 56). Investigations are crucial for identifying vulnerabilities in nuclear power plant operations, leading to legislative and engineering changes to prevent future accidents (55). Research on power plants and electrical systems has improved understanding of the mechanisms underlying nuclear disasters, highlighting aspects such as reactor design, maintenance, and violations of safety protocols (54).

This cluster’s focus on radiation injuries and biological materials indicates that research has also addressed the medical and biological consequences of radiation exposure (37, 39). Radiation injuries range from acute radiation sickness (ARS) to long-term health effects, including cancer and organ damage (38). This study investigates the physiological mechanisms underlying these injuries, focussing on the damage caused to DNA and cells due to increased radiation exposure (41, 42). The classification of occupational illnesses highlights the risks faced by workers in nuclear facilities, as exposure to radiation in the workplace has been linked to long-term health problems (40, 43).

Additionally, the terms health hazards and dosimetry suggest that researchers are improving the tools and models used to predict the effects of radiation exposure (44, 46). Accurate measurement of radiation exposure is crucial for public health and safety, enabling suitable medical interventions and minimizing long-term health risks for populations exposed to radiation (45).

#### 3.1.4 Yellow cluster: pediatric and long-term health implications

The yellow cluster highlights the enduring health effects of the CND, particularly for children and vulnerable populations (47, 48). The terms child, adolescent, newborn, and baby pertain to pediatric research, examining the health impacts of radiation exposure on younger populations (49, 50). Research demonstrates that children exposed to radiation have an increased risk of cancer, particularly thyroid neoplasms, due to the accumulation of radioactive iodine in their thyroid glands (51, 52).

Extended studies on cancer incidence, congenital anomalies, and radiation-induced malignancies reveal a troubling trend in the health outcomes of children exposed to radiation (53, 56). Including terminology such as retrospective research, follow-up studies, and risk variables stresses the necessity for ongoing surveillance of these populations, since the comprehensive consequences of radiation exposure may take decades to become apparent (54, 55). This has led to comprehensive research on the latency period for cancer progression and other chronic health conditions (37, 58).

Congenital abnormalities, pregnancy, and newborn status reflect concerns regarding the impact of radiation exposure on reproductive health and fetal development (38, 41). Studies indicate increased rates of congenital anomalies and developmental impairments in children of parents exposed to radiation (40, 42). The findings have important implications for public health, suggesting that the effects of radiation exposure may persist across multiple generations. This cluster highlights the importance of methodology and pathophysiology, advocating for research into the biological mechanisms that affect health outcomes to improve diagnostic and treatment strategies for impacted populations (43, 44).

The research on the CND is extensive, covering environmental, health, technological, and long-term pediatric dimensions. The network visualization demonstrates the interconnections among these issues, reflecting the complexity of the disaster’s impacts on human health, the environment, and nuclear safety regulations (45, 46). Each cluster offers significant insights into various facets of the catastrophe, highlighting the necessity for ongoing investigation and surveillance to comprehensively comprehend and alleviate the enduring repercussions of the CND (47, 48).

### 3.2 Relation to the UNSDGs

**Figure 3:**
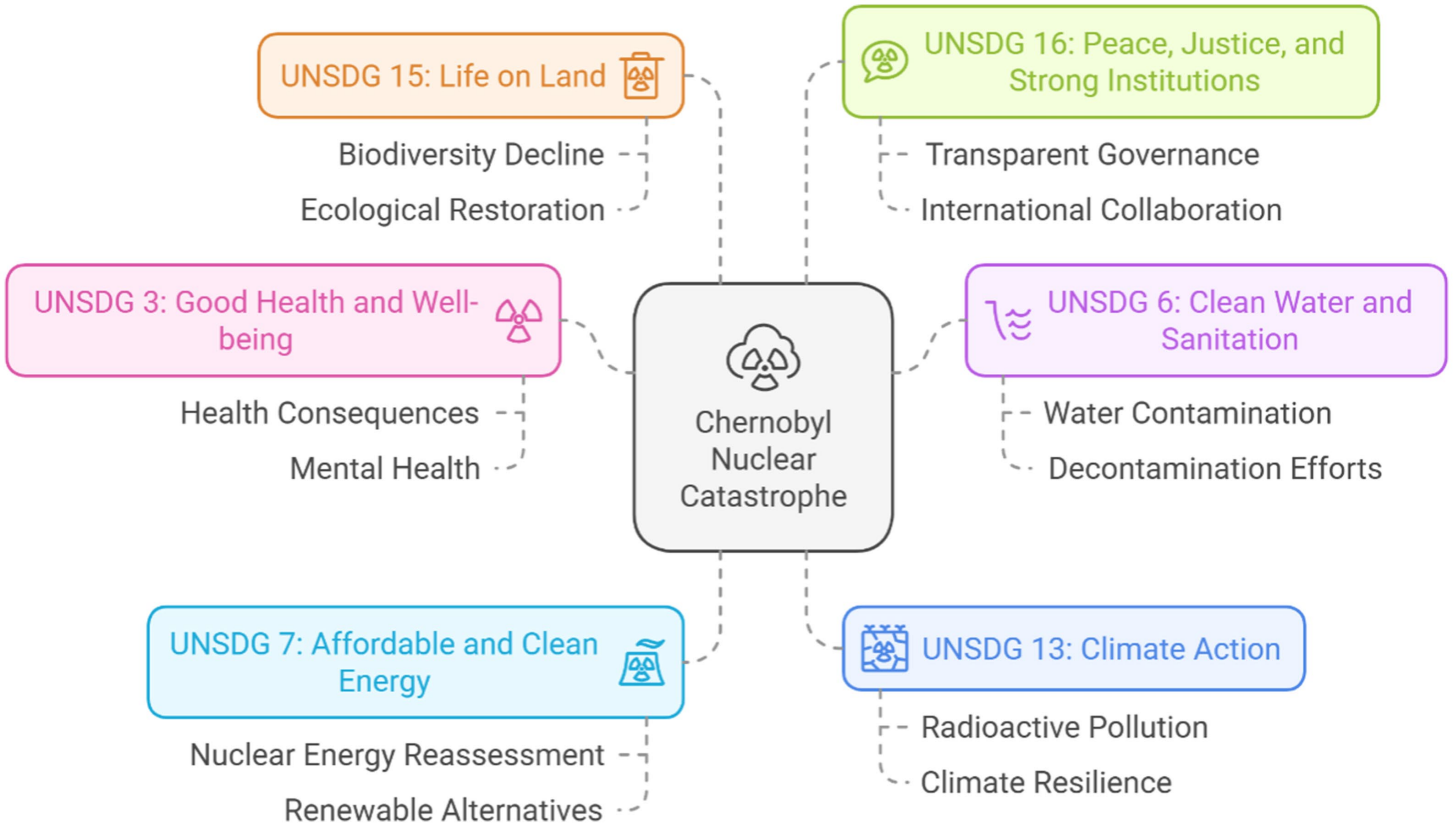
Overall relationships of Chernobyl nuclear disaster with United Nations’ Sustainable Development Goals, based on the present literature review.

The examination of the CND is highly relevant to numerous UNSDGs. The incident has significant implications for human health, the environment, energy policy, economic sustainability, and international collaboration, in accordance with the primary objectives of the UNSDGs (49, 50). This discussion addresses the pertinent UNSDGs related to the CND (Figure 3).

#### 3.2.1 UNSDG 3: good health and wellbeing

The health consequences of the CND, both immediate and long-term, are directly associated with UNSDG 3, which aims to ensure healthy lifestyles and improve wellbeing for individuals across all age groups (51, 52). The network visualization depicts clusters of research related to radiation-induced malignancies, thyroid neoplasms, and radiation injuries, all demonstrating the direct impact of nuclear accidents on public health (53, 56). Studies on thyroid cancer, mental health, post-traumatic stress disorder, and congenital malformations highlight the considerable effects of radiation exposure on individuals, especially within vulnerable populations such as children, women, and the older adult (54, 55).

The focus on long-term health monitoring, indicated by terms like follow-up research, retrospective study, and cancer incidence, suggests that sustaining good health and wellbeing requires continuous healthcare support for affected communities (37, 59). This emphasizes the necessity of investing in preventive healthcare initiatives and disaster preparedness to mitigate the long-term health effects of radiation exposure in potential future nuclear incidents (38, 41). Research on mental health disorders following disasters highlights the importance of psychological care and rehabilitation, especially for displaced populations, in alignment with UNSDG 3’s goal of addressing both physical and mental wellbeing (40, 42).

#### 3.2.2 UNSDG 6: clean water and sanitation

UNSDG 6 highlights the importance of clean water and sanitation, particularly in relation to the CND, due to the environmental pollution affecting water supplies (43, 44). The red cluster in the network visualization indicates radioactive contamination, soil pollution, and radioactive fallout, demonstrating that nuclear disasters pose a significant threat to the safety of water systems (45, 46). Following the CND, radioactive isotopes, including cesium-137 and iodine-131, impacted vast areas, notably water resources. This pollution negatively affects human populations and damages ecosystems, threatening food and water security in the impacted regions (47, 48).

The pollution of natural water bodies and the subsequent leaching of radioactive substances into groundwater threaten the achievement of UNSDG 6, which aims to ensure the availability and sustainable management of water for all (49, 50). Research on soil contamination and radioactive deposition demonstrates that decontamination and ecological restoration are essential for restoring safe water sources in regions impacted by nuclear disasters (51, 52). It is crucial to implement sustainable water management systems that account for potential environmental disasters to avert future occurrences (53, 56).

#### 3.2.3 UNSDG 7: affordable and clean energy

UNSDG 7 promotes access to affordable, reliable, sustainable, and modern energy sources. The CND had a substantial impact on global energy policy, particularly regarding the safety and sustainability of nuclear power (54, 55). The blue cluster in the network visualization emphasizes terms such as nuclear reactors, nuclear disasters, and power plants, reflecting the technical assessments and discourse surrounding the safety of nuclear energy (37, 58).

The CND underscores the risks associated with nuclear energy, prompting a reevaluation of its role in achieving clean energy goals (38, 41). Many countries re-evaluated their reliance on nuclear energy, choosing instead to pursue alternative renewable energy sources like solar and wind (40, 42). Nuclear energy is often considered a low-carbon energy source; however, it poses significant safety risks. The case of CND illustrates that the environmental and health impacts can greatly outweigh the benefits if safety protocols are not strictly enforced (43, 44). UNSDG 7 emphasizes that the provision of clean and affordable energy requires rigorous safety protocols and disaster preparedness to prevent nuclear incidents and reduce their impact on people and the environment (45, 46).

#### 3.2.4 UNSDG 13: climate action

UNSDG 13 requires prompt actions to tackle climate change and its effects. The CND highlights the environmental risks associated with nuclear power generation, which is viewed by some as a strategy to reduce greenhouse gas emissions (47, 48). The red cluster, which includes terms like radioactive pollution, radioactive fallout, and nuclear reactor accidents, underscores the paradox of nuclear energy: it offers a low-carbon alternative to fossil fuels while posing significant environmental risks in the event of accidents (49, 50).

The radioactive contamination of large areas in Ukraine, Belarus, and Russia has rendered these regions unsuitable for agriculture and human settlement, leading to challenges in land use and environmental restoration (51, 52). Addressing the climate change challenge requires a nuanced energy policy that considers the potential risks of nuclear energy in relation to its low-carbon benefits. Achieving UNSDG 13 requires the advancement of clean energy and the guarantee that energy systems are resilient to incidents and disasters, exemplified by the events at Chernobyl (53, 56).

#### 3.2.5 UNSDG 15: life on land

The red and yellow clusters illustrate the significant environmental and ecological damage caused by the CND, directly correlating with UNSDG 15, which focusses on the protection, restoration, and sustainable use of terrestrial ecosystems (54, 55). The catastrophe resulted in significant radioactive contamination, as evidenced by the concepts of radioactive pollution, radioactive fallout, and contamination. The contaminants adversely affected the nearby soil, vegetation, and wildlife, leading to substantial alterations in ecosystems and a decline in biodiversity in the affected regions (37, 59).

Studies on soil contamination and radiation-induced changes in ecosystems demonstrate that extended remediation of these environments is essential for achieving Life on Land (38, 41). Contaminated areas like the Chernobyl Exclusion Zone continue to exhibit altered biological dynamics, characterized by the extinction of some species and the proliferation of others, such as wolves and wild boars, due to the absence of human activity (40, 42). Achieving UNSDG 15 requires ongoing efforts to monitor and rehabilitate ecosystems to restore biodiversity and mitigate the persistent environmental damage caused by radioactive pollution (43, 44).

#### 3.2.6 UNSDG 16: peace, justice, and strong institutions

UNSDG 16 emphasizes the promotion of peaceful and inclusive societies, equitable justice access, and strong institutions. The CND, particularly in relation to the geopolitical aspects of the red cluster (e.g., Belarus, Ukraine, disasters, and radioactive contamination), illustrates the importance of transparent governance, international cooperation, and effective institutional responses to crises (45, 46). The catastrophe exposed significant shortcomings in official response systems, particularly in the Soviet Union, where initial concealment of information exacerbated the public health crisis and delayed foreign aid (47, 48).

To ensure peace, justice, and strong institutions in the context of nuclear disasters, governments must adopt transparent policies, maintain open communication with the public, and collaborate with international organizations (49, 50). The CND emphasized the importance of risk assessment, radiation monitoring, and effective emergency response systems in preventing fatalities and mitigating environmental damage (51, 52). Establishing strong institutions that can effectively manage nuclear safety, environmental conservation, and public health is essential for preventing future disasters of similar scale (53, 56).

The legacy of the CND offers important insights into various dimensions of sustainability, encompassing health and environmental restoration, institutional governance, and energy policy. The ongoing relevance of the catastrophe in discussions regarding the UNSDGs highlights the continued need to understand and address the long-term effects of nuclear accidents (54, 55). The lessons from Chernobyl will be crucial in achieving a balance among energy security, environmental stewardship, and human welfare as the world progresses toward a sustainable future (37, 59).

### 3.3 Synthesis and future challenges

The CND serves as a critical example of the substantial environmental, health, and socio-political consequences of industrial accidents (23, 25). The legacy of Chernobyl is highly relevant to multiple UNSDGs, even though these goals were established decades later. The impact of the disaster on public health aligns with the goals of UNSDG 3 (Good Health and Wellbeing), emphasizing the need for ongoing health monitoring and comprehensive healthcare interventions, particularly regarding radiation-related illnesses (26, 60). The pollution of aquatic environments and ecosystems highlights the urgent need for UNSDG 6 (Clean Water and Sanitation) and UNSDG 15 (Life on Land), which underscore the significance of sustainable resource management and ecosystem restoration (50, 52). The impact of Chernobyl on global energy discourse is fundamentally associated with UNSDG 7 (Affordable and Clean Energy), which promotes the equilibrium between energy needs and safety (24, 61).

The paradox of ecosystem recovery in the exclusion zone, where wildlife has flourished in the absence of human interference despite radiation, underscores complex insights regarding UNSDG 13 (Climate Action) and ecosystem resilience (13, 62). The unintentional restoration of the region demonstrates nature’s ability to recover without human intervention; however, the persistent radiation highlights the long-lasting and intricate consequences of industrial disasters (58, 63). UNSDG 17 (Partnerships for the Goals) is relevant to the global response and cooperation necessary to mitigate the effects of Chernobyl. The catastrophe prompted global efforts to improve nuclear safety protocols and foster international collaboration, a crucial consideration for the internationalization of the UNSDGs (53, 56).

### 3.4 Prospective challenges and dilemmas

**Figure 4:**
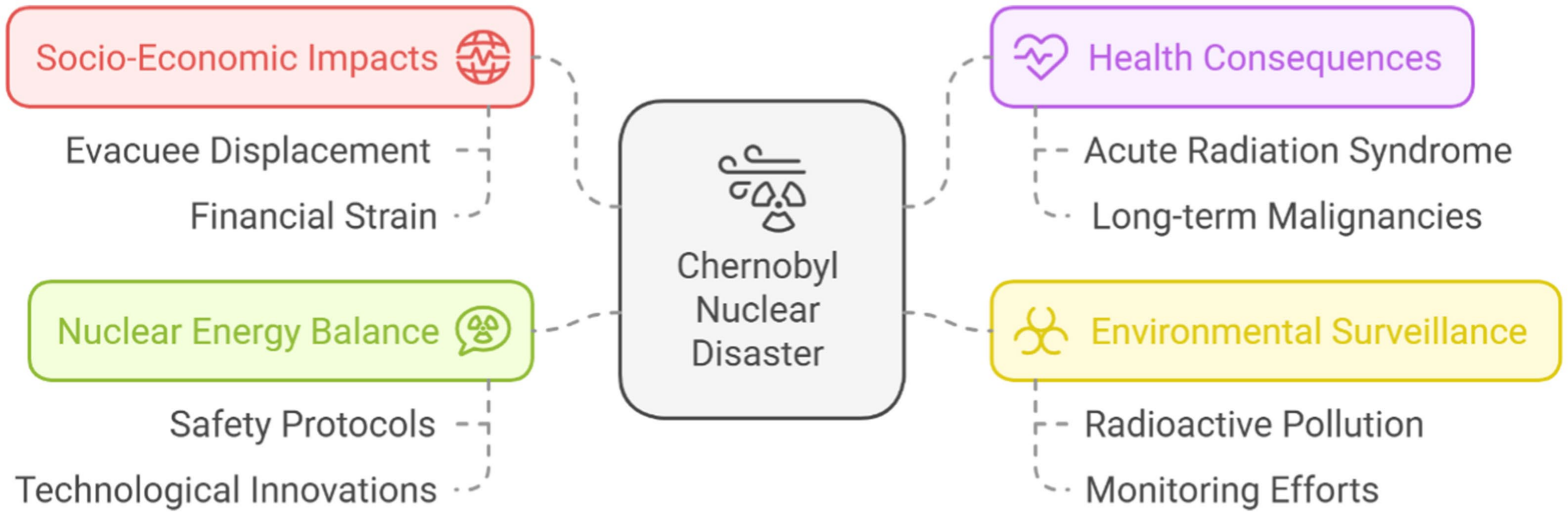
Overall prospective challenges and dilemmas from Chernobyl nuclear disaster based on the present literature review.

This analysis provides a comprehensive examination of these impacts based on the available references (Figure 4).

#### 3.4.1 Equilibrating nuclear energy and safety

Nuclear power continues to be a debated alternative in the pursuit of low-carbon energy solutions (51, 64). The CND underlined the significant risks linked to nuclear energy, prompting numerous countries to decrease or abandon their dependence on it. Nuclear energy remains essential as a low-emission energy source in light of the pressing need to combat climate change (52, 65). The challenge involves reconciling the need for clean energy with the associated risks of CND. Advancements in nuclear technology, including next-generation reactors equipped with passive safety systems, are essential for risk mitigation; however, they necessitate substantial investment and international collaboration (57, 66). This prompts a discussion regarding the prioritization of rapid advancements in renewable energy technologies versus nuclear power, taking into account the relevant trade-offs.

The CND significantly influenced the global nuclear industry, diminishing public trust in nuclear energy and leading to an international re-evaluation of safety protocols (67). Some countries reduced their dependence on nuclear power, whereas others, such as Russia, continued to expand their nuclear capabilities with improved safety protocols (68). The disaster prompted advancements in reactor design and the establishment of enhanced safety frameworks globally (69).

#### 3.4.2 Socio-economic impact

The CND had significant socio-economic impacts, affecting around 350,000 evacuees and placing considerable pressure on the social and economic frameworks of the impacted areas (70). Ukraine allocates 5–7% of its annual government budget to address the long-term consequences of the disaster, indicating a persistent financial burden (71). Significant economic losses resulting from compensation, healthcare, and cleanup initiatives have prompted changes in regional energy policies (68). The disaster has resulted in enduring socio-economic decline and significant psychological consequences, such as increased stress and diminished quality of life for the impacted populations (72, 73).

The health consequences of CND are both immediate and long-term. Acute radiation syndrome resulted in the deaths of 28 emergency workers, with a total of 64 fatalities directly associated with the disaster (70, 74). Longitudinal studies indicate increased incidences of thyroid cancer, leukemia, and other malignancies, especially in children exposed to radiation (75). Mental health disorders such as anxiety, depression, and post-traumatic stress disorder are prevalent public health issues affecting survivors and workers (73). Documented secondary health effects include cardiovascular diseases, cataracts, and endocrine disorders among individuals exposed to lower radiation doses (75).

#### 3.4.3 Long-term environmental and health surveillance

A fundamental insight from the CND is the necessity for ongoing, long-term monitoring of environmental and public health effects (61, 76). The enduring presence of radioactive pollution, attributed to isotopes with half-lives extending over decades, underscores the continuous risk to the area. This issue affects countries dealing with nuclear contamination, requiring significant investment in long-term monitoring and remediation strategies (23, 47). The high costs and complexities of such initiatives frequently strain resources, thereby complicating the prioritization of objectives. Monitoring radiation-induced health effects, including malignancies, necessitates ongoing investment in healthcare infrastructure and research, presenting considerable challenges for resource-limited countries (50, 62).

The environmental impact of CND was extensive and multifaceted. The release of radioactive materials contaminated land, water, and air throughout Europe, significantly impacting Belarus, Ukraine, and Russia (77, 78). The 30-kilometer exclusion zone surrounding the reactor is uninhabitable; however, it has unintentionally transformed into a wildlife sanctuary due to the lack of human presence, despite ongoing high radiation levels (70, 79). Although biodiversity experienced significant declines, certain species have adapted to the radioactive conditions, while others display genetic mutations (77). Additionally, radioactive isotopes have contaminated water systems, including significant rivers in Ukraine, presenting enduring risks to human and ecological health (80). The persistent environmental challenges highlight the need for continuous monitoring and global cooperation in tackling nuclear contamination.

#### 3.4.4 International governance and accountability

The CND emphasized the importance of international cooperation in nuclear safety management and disaster response (47, 48). It is essential to develop robust global governance frameworks to prevent such tragedies. There are challenges regarding the assurance of equal accountability (15, 23). Certain nations have the scientific capacity to enhance nuclear safety, while others may lack the necessary resources or political will to achieve this (81, 82). Global governance processes must be inclusive, transparent, and effectively enforced. Accountability issues, particularly in relation to historical nuclear disasters, remain a significant obstacle to compensation and justice for affected communities (58, 64, 83–88).

The absence of a cohesive international framework for nuclear liability and disaster response hinders efforts to establish accountability and provide equitable support to affected nations (89–91). Disparities in financial responsibility, legal obligations, and enforcement mechanisms can impede collaborative progress and intensify tensions among nations with varying nuclear capabilities (48, 81). Establishing standardized protocols for nuclear disaster preparedness, compensation, and remediation is essential, supported by multilateral agreements and a strong enforcement mechanism. Enhancing the IAEA’s role in compliance oversight and collaboration may address existing gaps and improve global nuclear safety (92–95).

#### 3.4.5 Resilience against climate change

The ecological restoration in the Chernobyl Exclusion Zone offers important insights into resilience while also underscoring the difficulties of executing effective environmental protection strategies amid climate change (52, 96, 97). The rising incidence of natural disasters, industrial accidents, and ecosystem degradation associated with climate change necessitates that recovery efforts be sustainable and adaptable (47, 48, 98, 99). The main challenge is achieving a balance between human development and environmental conservation. Industrialized nations frequently emphasize energy and industrial development, whereas developing countries may be constrained by insufficient resources to support recovery efforts (57, 76). The disparity in resilience capacity poses substantial global challenges to climate change mitigation and disaster risk reduction, as indicated in the UNSDGs (56, 66).

The restoration efforts in Chernobyl underline the necessity for interdisciplinary approaches that combine ecological science, socio-economic policies, and technological innovation. Collaborative frameworks are crucial for tackling complex challenges, including radioactive contamination, biodiversity loss, and land-use planning in areas susceptible to disasters. Global partnerships and knowledge-sharing enable both industrialized and developing nations to improve their resilience strategies, aligning recovery efforts with climate adaptation goals and equitable resource distribution (48, 53). This approach supports long-term ecological sustainability and promotes a unified response to global climate-induced challenges.

#### 3.4.6 Ethical quandaries in energy policy

Nuclear energy poses ethical challenges due to potential risks to human life and the environment (40, 53). Nuclear energy can meet the global demand for clean energy; however, the risk of catastrophic events, exemplified by Chernobyl, necessitates an assessment of whether the benefits outweigh the risks (59, 76). The management of nuclear waste, which poses risks for thousands of years, complicates energy policy decisions (18, 19). Policymakers face the ethical challenge of reconciling the immediate benefits of nuclear energy with its long-term environmental and health impacts, while also considering the responsibility of managing nuclear waste for future generations (22, 83, 100).

The CND serves as a critical case study for understanding the complex interplay among energy policy, environmental management, and public health (24, 45, 101). The global quest for sustainable solutions to climate change and energy requirements underscores the critical lessons from Chernobyl regarding the intrinsic risks and challenges linked to nuclear power (25, 62, 102). Balancing the demand for clean energy with safety, resilience, and ethical governance will continue to be a critical global challenge in the coming decades (24, 53).

## 4 Conclusion

The current assessment findings could provide crucial insights for advancing the UNSDGs, particularly in health, energy, climate action, and international collaboration. The global community must prioritize strong governance institutions, continuous environmental and health monitoring, and ethical energy policymaking. Achieving a balance between clean energy requirements and safety is critical, as is ensuring long-term ecosystem resilience and encouraging cross-border collaboration. The legacy of CND emphasizes the ongoing need for attention and innovation to prevent future nuclear tragedies while promoting sustainability. As a result, the CND has previously highlighted the link between human health, environmental sustainability, and institutional governance. This is required to reduce the long-term consequences of nuclear accidents. This may necessitate a comprehensive plan that includes environmental restoration, public health interventions, and a strong energy policy. The CND has sparked global discussion about the role of nuclear energy in sustainable development, imparting important lessons relevant to the UNSDGs. It is envisaged that incorporating the current findings into policy frameworks will result in a safer, more sustainable future while reducing the risks connected with nuclear technologies.

## Data Availability

The original contributions presented in the study are included in the article/supplementary material, further inquiries can be directed to the corresponding authors.

## References

[1] Bihar J. The Chernobyl disaster [die Tschernobyl-Katastrophe]. Geogr Rundsch. (2021) 73:56.

[2] Min J. Field disasters, routine shifts, and adaptation performance: evidence from the Chernobyl disaster. Organ Stud. (2024) 45:137–60. doi: 10.1177/01708406221124795

[3] Rogovskyy AS Threadgill DW Akimov IA Nebogatkin IV Rogovska YV Melnyk MV . Borrelia and other zoonotic pathogens in *Ixodes ricinus* and *Dermacentor reticulatus* ticks collected from the Chernobyl exclusion zone on the 30th anniversary of the nuclear disaster. Vect Borne Zoonot Dis. (2019) 19:466–73. doi: 10.1089/vbz.2018.2318, PMID: 31112094

[4] Shehadeh HA. Chernobyl disaster optimizer (CDO): a novel meta-heuristic method for global optimization. Neural Comput Applic. (2023) 35:10733–49. doi: 10.1007/s00521-023-08261-1

[5] Donstrup M Algaba C. The iron screen: an ideological analysis of the discourse on Russia through the nuclear disaster of Chernobyl. J Cult Res. (2020) 24:253–68. doi: 10.1080/14797585.2020.1810584

[6] Hancock S Vo NTK Goncharova RI Seymour CB Byun SH Mothersill CE. One-decade-spanning transgenerational effects of historic radiation dose in wild populations of bank voles exposed to radioactive contamination following the Chernobyl nuclear disaster. Environ Res. (2020) 180:108816. doi: 10.1016/j.envres.2019.108816, PMID: 31627157

[7] Trishchynska MA Onopriyenko OP. Epidemiology of stroke in the left bank area of Kyiv region on the eve of the 38th anniversary of the man-made Chernobyl disaster: implementing international experience into practice (modern view of the problem and own research). Int Neurol J. (2024) 20:110–8. doi: 10.22141/2224-0713.20.2.2024.1058

[8] Cucu AI Costea CF Carauleanu A Dumitrescu GF Sava A Scripcariu IS . Meningiomas related to the Chernobyl irradiation disaster in North-Eastern Romania between 1990 and 2015. Rev Chim. (2018) 69:1562–5. doi: 10.37358/rc.18.6.6369

[9] Erolova Y Tsyryapkina Y. Local reflections on the Chernobyl disaster 35 years later: peripheral narratives from Ukraine, Belarus, Russia, and Bulgaria. Comp South Eur Stud. (2023) 71:12–31. doi: 10.1515/soeu-2021-0069

[10] Nakamura T Lloyd S Maruyama A Masuda S. Public reaction to disaster reconstruction policy: case studies of the Fukushima and Chernobyl nuclear accidents. J Disaster Res. (2021) 16:1207–33. doi: 10.20965/jdr.2021.p1207

[11] Maslov VP. On mathematical investigations related to the Chernobyl disaster. Russ J Math Phys. (2018) 25:309–18. doi: 10.1134/S1061920818030044

[12] Reste J Zvagule T Kurjane N Šķesters A Silova A Eglite M . Investigations on health conditions of Chernobyl nuclear power plant accident recovery workers from Latvia in late period after disaster. Proc Latvian Acad Sci B. (2016) 70:257–65. doi: 10.1515/prolas-2016-0040

[13] Slusky DA Cwikel J Quastel MR. Chronic diseases and mortality among immigrants to Israel from areas contaminated by the Chernobyl disaster: a follow-up study. Int J Public Health. (2017) 62:463–9. doi: 10.1007/s00038-017-0941-1, PMID: 28130563

[14] Zhukova E. Foreign aid and identity after the Chernobyl nuclear disaster: how Belarus shapes relations with Germany, Europe, Russia, and Japan. Coop Confl. (2017) 52:485–501. doi: 10.1177/0010836717710529

[15] Penubarthi SR Korrapati RR Janamala V Nimmagadda C Veerendra AS Ravindrakumar S. Chernobyl disaster optimizer-based optimal integration of hybrid photovoltaic systems and network reconfiguration for reliable and quality power supply to nuclear research reactors. Modelling. (2024) 5:1268–85. doi: 10.3390/modelling5030065

[16] Hovhannisyan NM Karapetyan AG Grigoryan VS. The assessment of cytogenetic indices of Chernobyl power plant disaster liquidators. RAD Conf Proc. (2017) 2:1–5. doi: 10.21175/RadProc.2017.01

[17] Janiak MK Kamiński G. Thyroid cancer in regions most contaminated after the Chernobyl disaster. J Biomed Phys Eng. (2024) 14:299–308. doi: 10.31661/jbpe.v0i0.2402-1722, PMID: 39027710 PMC11252555

[18] Körblein A. Letter to the editor of Heliyon re: De novo congenital malformation frequencies in children from the Bryansk region following the Chernobyl disaster (2000–2017). Heliyon. (2020) 6:e04616. doi: 10.1016/j.heliyon.2020.e04616 32885067 PMC7452506

[19] Lopes ARS Rodrigues RR. Industrialization and environmental crisis: a representation of the nuclear disaster in Chernobyl prayer by Svetlana Aleksievich. Temp Argument. (2019) 11:44–66. doi: 10.5965/2175180311262019044

[20] Zhukova E. Nuclear disaster as chronic crisis: accounts of radiation embodiment by survivors of the Chernobyl nuclear disaster from Belarus born before, in, and after 1986. Health. (2020) 24:589–605. doi: 10.1177/1363459319829190, PMID: 30755050

[21] Volosovets O Kryvopustov S Beketova G Volosovets A Savinova K. Comparison of the incidence of cardiovascular diseases in children living in areas that were contaminated by the Chernobyl disaster and in other ecologically unfavorable regions of Ukraine (results of 24-year monitoring). Kardiol Belarusi. (2020) 12:169–77. doi: 10.34883/PI.2020.12.2.003

[22] Feshchuk Y Nizhnyk V Nekora V Teslenko O. Improving the system for responding to fire in areas contaminated by the Chernobyl disaster. News Natl Acad Sci Repub Kazak Series Geol Tech Sci. (2022) 1:152–8. doi: 10.32014/2022.2518-170X.152

[23] Aribowo W Shehadeh HA. Novel modified Chernobyl disaster optimizer for controlling DC motor. Indon Jo Electr Eng Comput Sci. (2024) 35:1361–9. doi: 10.11591/ijeecs.v35.i3.pp1361-1369

[24] Ludovici GM Oliveira de Souza S Chierici A Cascone MG d’Errico F Malizia A. Adaptation to ionizing radiation of higher plants: from environmental radioactivity to Chernobyl disaster. J Environ Radioact. (2020) 222:106375. doi: 10.1016/j.jenvrad.2020.106375, PMID: 32791372

[25] Datta D Nandi A. “What is the cost of lies?” historiography of a disaster and the collapse of the soviet metanarrative in Craig Mazin and Johan Renck’s HBO miniseries Chernobyl. Univ Buchar Rev. (2022) 12:60–9. doi: 10.31178/UBR.12.2.5

[26] Kovalenko BS Golivets TP Volkov DV Kovalenko IB Zakharov OV. Multipurpose epidemiological survey of multiple primary malignancies at the territories affected by Chernobyl disaster. Int J Pharm Technol. (2016) 8:14307–16.

[27] IAEA. The international Chernobyl project International Atomic Energy Agency (1991). Vienna, Austria: International Atomic Energy Agency

[28] IAEA. One decade after Chernobyl: Summing up the consequences of the accident International Atomic Energy Agency (1996). Vienna, Austria: International Atomic Energy Agency

[29] IAEA. Environmental consequences of the Chernobyl accident and their remediation: twenty years of experience. Radiological assessment reports series no. 8 International Atomic Energy Agency (2006). Vienna, Austria: International Atomic Energy Agency

[30] IAEA. Chernobyl: Looking back to go forward. Proceedings Series International Atomic Energy Agency (2008). Vienna, Austria: International Atomic Energy Agency

[31] Van Eck NJ Waltman L. Software survey: VOSviewer, a computer program for bibliometric mapping. Scientometrics. (2010) 84:523–38. doi: 10.1007/s11192-009-0146-3, PMID: 20585380 PMC2883932

[32] Moher D Liberati A Tetzlaff J Altman DG. PRISMA group preferred reporting items for systematic reviews and meta-analyses: the PRISMA statement. PLoS Med. (2009) 6:e1000097. doi: 10.1371/journal.pmed.1000097, PMID: 19621072 PMC2707599

[33] Scopus. (2024). Scopus: comprehensive, multidisciplinary, trusted abstract and citation database. Available at: https://www.elsevier.com/products/scopus (Accessed December 26, 2024).

[34] Van Eck NJ Waltman L. Visualizing bibliometric networks In: Ding Y Rousseau R Wolfram D, editors. Measuring scholarly impact: methods and practice. Dordrecht, Netherlands: Springer (2014). 285–320.

[35] Waltman L Van Eck NJ Noyons EC. A unified approach to mapping and clustering of bibliometric networks. J Informet. (2010) 4:629–35. doi: 10.1016/j.joi.2010.07.002

[36] Van Eck NJ Waltman L. Citation-based clustering of publications using CitNetExplorer and VOSviewer. Scientometrics. (2017) 111:1053–70. doi: 10.1007/s11192-017-2300-7, PMID: 28490825 PMC5400793

[37] Fechter AM. Humanitarianism, mobility and kinship: a reply to ‘chronic crisis and nuclear disaster humanitarianism: recuperation of Chernobyl and Fukushima children in Italy’ by Ekatherina Zhukova. Glob Discourse. (2022) 12:638–40. doi: 10.1332/204378921X16321295120732

[38] Jargin SV. Debate on the Chernobyl disaster: response to Alison Rosamund Katz. Int J Health Serv. (2017) 47:150–9. doi: 10.1177/0020731416679343, PMID: 27956579

[39] Zhukova E. Private humanitarian responses to disaster vulnerabilities: the Chernobyl children from Belarus in Italy. Childhood. (2020) 27:238–53. doi: 10.1177/0907568220901747

[40] Havenaar JM Bromet EJ Gluzman S. The 30-year mental health legacy of the Chernobyl disaster. World Psychiatry. (2016) 15:181–2. doi: 10.1002/wps.20335, PMID: 27265712 PMC4911770

[41] Marešová D Hanslík E Juranová E Sedlářová B. Case study: Long-term consequences of atmospheric tests of nuclear weapons and Chernobyl disaster on the territory of South Bohemia (Czech Rep ublic). In Peterson M, editor. *The Chernobyl disaster*. USA: Nova Science Publishers (2016). pp. 103–133.

[42] Tzoumis KA. Chernobyl disaster (1986). Toxic Chem Am. (2020) 1-2:101–6.

[43] Aitsi-Selmi A Murray V. The Chernobyl disaster and beyond: implications of the Sendai framework for disaster risk reduction 2015–2030. PLoS Med. (2016) 13:e1002017. doi: 10.1371/journal.pmed.1002017, PMID: 27111030 PMC4844113

[44] Schmid S. Chernobyl: data wars and disaster politics. Nature. (2019) 566:450–1. doi: 10.1038/d41586-019-00678-w

[45] Cocco G. The disaster of the Doce River, the Brazilian Chernobyl [La catastrophe du rio Doce, le Tchernobyl brésilien]. Multitudes. (2016) 62:5–13. doi: 10.3917/mult.062.0005, PMID: 18052372

[46] Lindbladh J. The Chernobyl disaster: from the explosion to the closing of the plant. Baltic Worlds. (2019) 12:101–3.

[47] Okada T Cholii S Karácsonyi D Matsumoto M. Communities in Fukushima and Chernobyl—enabling and inhibiting factors for recovery in nuclear disaster areas. In Karácsonyi, D, Taylor, A, Bird, D, editors. The demography of disasters: Impacts for population and place. Switzerland: Springer. (2020), pp. 211–32., PMID: 39944376

[48] Szalai S Farkas N Veszpremi B Bodis J Kovacs K Farkas B. Assessment of the potential impacts of the Chernobyl nuclear disaster on maternal and fetal health in Hungary. J Matern Fetal Neonatal Med. (2022) 35:9481–8. doi: 10.1080/14767058.2022.2044471, PMID: 35240917

[49] Danka I Tanács J. Rationalising rule violation in the case of the Chernobyl disaster: six systematic excuses. Infor Tarsad. (2021) 21:19–37. doi: 10.22503/inftars.XXI.2021.4.2

[50] Romanovich IK Bruk GY Bazyukin AB Bratilova AA Yakovlev VA. The dynamics of the average annual and cumulative radiation exposure doses of the adult population of the Russian Federation after the Chernobyl disaster. Public Health Life Environ. (2020) 2020:33–8. doi: 10.35627/2219-5238/2020-324-3-33-38

[51] Kékesdi-Boldog D. The Chernobyl disaster: a case study on the information policy of the Kádár regime. Central Eur J Commun. (2019) 12:78–91. doi: 10.19195/1899-5101.12.1(22).5

[52] Yushkova E. Contribution of transposable elements to transgenerational effects of chronic radioactive exposure of natural populations of *Drosophila melanogaster* living for a long time in the zone of the Chernobyl nuclear disaster. J Environ Radioact. (2022) 251-252:106945. doi: 10.1016/j.jenvrad.2022.106945 35696883

[53] Kempin S Finger PT Gale RP Rescigno J Rubin J Choi W . A cluster of vitreoretinal lymphoma in New York with possible link to the Chernobyl nuclear disaster. Leuk Lymphoma. (2018) 59:1998–2001. doi: 10.1080/10428194.2017.1403025, PMID: 29164983 PMC6082125

[54] Ager AA Lasko R Myroniuk V Zibtsev S Day MA Usenia U . The wildfire problem in areas contaminated by the Chernobyl disaster. Sci Total Environ. (2019) 696:133954. doi: 10.1016/j.scitotenv.2019.133954

[55] Meshkov NA. Pathogenesis of cardiovascular diseases in liquidators of Chernobyl disaster in the long term. Radiat Risk. (2016) 25:73–85. doi: 10.21870/0131-3878-2016-25-3-73-85

[56] Podsonnaya IV Shumacher GI Efremushkin GG Gelobetskaya ED. Formation of paroxysmal brain activity in the liquidators of the consequences of the Chernobyl nuclear disaster. Zhurnal Nevrologii i Psihiatrii imeni S.S. Korsakova. (2015) 115:71–6. doi: 10.17116/jnevro201511510171-76, PMID: 26525626

[57] Peterson M. (Ed.). (2016). The Chernobyl disaster. The Chernobyl disaster. USA: Nova Science Publishers.

[58] Zhukova E. Chronic crisis and nuclear disaster humanitarianism: recuperation of Chernobyl and Fukushima children in Italy. Glob Discourse. (2022) 12:616–37. doi: 10.1332/204378921X16320401719127

[59] Zhukova E. Kinning as intimate disaster response: from recuperation in host families to educational migration of the Chernobyl children from Belarus to Italy. Identities. (2022) 29:205–22. doi: 10.1080/1070289X.2019.1686877

[60] Korsakov AV Lagerev DG Pugach LI Troshin VP Geger EV Titarev DV. The application of visual analytics methods to analyze the dynamics of stillbirth in radiation-contaminated areas of the Bryansk region after the Chernobyl disaster (1986-2016). CEUR Workshop Proc. (2019) 2485:86–91. doi: 10.30987/graphicon-2019-2-86-91

[61] Legeza VI Stepan GG Zagorodnikov GG Reznik VM Aksenova NV. Major life expectancy risks in the military liquidators of the Chernobyl disaster in 1986. Medico-biological and socio-psychological issues of safety in emergency situations. (2024) 2:39–48. doi: 10.25016/2541-7487-2024-0-2-39-48

[62] Takahashi S Suchara I Okamoto K Sucharová J Umegaki K Fujiyoshi R. Retention of 137Cs in forest floor at three temperate coniferous forest stands in the Czech Republic diversely affected by fallout after the Chernobyl disaster in 1986. J Radioanal Nucl Chem. (2017) 311:929–35. doi: 10.1007/s10967-016-5048-2

[63] Rozhko AV. The experience of the republican research Centre for Radiation Medicine and Human Ecology in the implementation of the union state measure to provide comprehensive medical care to the population affected by the Chernobyl disaster. Radiatsion Gygiena. (2022) 15:110–5. doi: 10.21514/1998-426X-2022-15-3-110-115

[64] Vettenranta S. Crisis communication and the Norwegian authorities: 22 July and the Chernobyl disaster: two catastrophes, dissimilar outcomes. Nordicom Rev. (2015) 36:51–64. doi: 10.1515/nor-2015-0005

[65] Korsakov AV Geger EV Lagerev DG Pugach LI Pivovarov YP Korolik VV . Comparative analysis of the prevalence of congenital malformations of the brain in children of the radioactively contaminated territories of the Bryansk region after the Chernobyl disaster (1999-2014). Gigiena Sanitariya. (2020) 99:356–62. doi: 10.47470/0016-9900-2020-99-4-356-362

[66] Bolt MA Helming LM Tintle NL. The associations between self-reported exposure to the Chernobyl nuclear disaster zone and mental health disorders in Ukraine. Front Psych. (2018) 9:32. doi: 10.3389/fpsyt.2018.00032, PMID: 29497388 PMC5818457

[67] Sihver L Yasuda N. Causes and radiological consequences of the Chernobyl and Fukushima nuclear accidents. J Nucl Eng Radiat Sci. (2018) 4:020914. doi: 10.1115/1.4037116

[68] Popova LV. Russian energy policy - twenty years after Chernobyl: no lessons learned? Energy Environ. (2006) 17:417–37. doi: 10.1260/095830506778119461

[69] Stsiapanau A. Nuclear exceptionalism in the former Soviet Union after Chernobyl and Fukushima In: Stoetzer M Schlüter A Veloso ALT, editors. The Fukushima effect: A new geopolitical terrain. London, UK: Taylor & Francis/Routledge (2015). 121–40.

[70] Cardis E Hatch M. The Chernobyl accident—an epidemiological perspective. Clin Oncol. (2011) 23:251–60. doi: 10.1016/j.clon.2011.01.510, PMID: 21396807 PMC3107017

[71] Bertell R. Chernobyl: an unbelievable failure to help. Int J Health Serv. (2008) 38:543–60. doi: 10.2190/HS.38.3.i, PMID: 18724581

[72] Balonov M. Third annual Warren K. Sinclair keynote address: retrospective analysis of impacts of the Chernobyl accident. Health Phys. (2007) 93:383–409. doi: 10.1097/01.HP.0000282109.20364.37, PMID: 18049216

[73] Bromet EJ Havenaar JM Bromet EJ Havenaar JM. The long-term mental health impacts of the Chernobyl accident In: Neria Y Galea S Norris FH, editors. Mental health and disasters. Cambridge, UK: Cambridge University Press (2009). 441–53.

[74] Sumner D. Health effects resulting from the Chernobyl accident. Med Confl Surviv. (2007) 23:31–45. doi: 10.1080/13623690601084583 17370857

[75] Zablotska LB. 30 years after the Chernobyl nuclear accident: time for reflection and re-evaluation of current disaster preparedness plans. J Urban Health. (2016) 93:407–13. doi: 10.1007/s11524-016-0053-x, PMID: 27130482 PMC4899336

[76] Orsatti G. Government R&D and green technology spillovers: the Chernobyl disaster as a natural experiment. J Technol Transfer. (2024) 49:581–608. doi: 10.1007/s10961-023-10000-6

[77] Anspaugh L. Environmental consequences of the Chernobyl accident and their remediation: 20 years of experience. Chernobyl. (2008) 47:141–4.

[78] Balonov M Bouville A. Radiation exposures due to the Chernobyl accident In: Elvers JFD, editor. Encyclopedia of environmental health. Amsterdam, Netherlands: Elsevier (2019). 709–20.

[79] Kumar BR. Case 35: Chernobyl new safe confinement project In: Project finance: structuring, valuation and risk management for major projects. Cham: Springer International Publishing (2022). 263–5.

[80] Vengosh A. Rooting out radioactive groundwater. Geotimes. (2006) 51:18–21.

[81] Hoensch V. The Chernobyl, Fukushima Daiichi, and Deepwater Horizon disasters froma natural science and humanities perspective. Berlin, Germany: Springer-Verlag (2022). p. 199.

[82] Radziuk H Shapsheeva T. Application of agronomical approaches to rehabilitating territories of the Republic of Belarus affected by the Chernobyl disaster. Environ Sci Pollut Res. (2020) 27:8003–15. doi: 10.1007/s11356-019-07456-1, PMID: 31893364

[83] Chuchvaha H. Memory, trauma, and the maternal: post-apocalyptic view of the Chernobyl/Chornobyl/Charnobyl nuclear disaster. East/west. J Ukrain Stud. (2020) 7:3–31. doi: 10.21226/ewjus608

[84] Aleksanin SS Rybnikov VY Rogalev KK Tarita VA. Specialized medical care in a round-the-clock hospital for citizens exposed to radiation as a result of the Chernobyl disaster. Med Biol Soc Psychol Issues Saf Emerg Situat. (2019) 4:5–11. doi: 10.25016/2541-7487-2019-0-4-05-11

[85] Bakota D Machowski R Płomiński A Ramanchuk A Rzętała M Zastavetska L. The disaster as a factor in the development of modern tourism: a study case based on the Chernobyl nuclear power plant. J Environ Manag Tour. (2020) 11:1729–41. doi: 10.14505//jemt.11.7(47).14

[86] Bakota D Zastavetska L Płomiński A. The disaster in Chernobyl nuclear power plant and tourism: condition of and prospects for the development of tourism in the area of radioactive contamination [Katastrofa w czarnobylskiej elektrowni jądrowej a turystyka. Stan i perspektywy rozwoju turystyki na obszarze skażenia promieniotwórczego]. Rocznik Ochrona Środowiska. (2018) 20:495–511.

[87] Belova YY Muravitskaia ME Melnikova NM. Collective trauma and the memory of the accident at the Chernobyl nuclear power plant: 35 years after the disaster. Etnograficheskoe Obozrenie. (2022) 2022:197–218. doi: 10.31857/S0869541522030113

[88] Büntgen U Jäggi M Egli S Heule M Peter M Zagyva I . No radioactive contamination from the Chernobyl disaster in Hungarian white truffles (tuber magnatum). Environ Pollut. (2019) 252:1643–7. doi: 10.1016/j.envpol.2019.06.108, PMID: 31284206

[89] Ernst T Rinke J Hagen J Dmytrenko I Hochhaus A Dyagil I. Molecular-defined clonal evolution in patients with chronic myeloid leukemia who were exposed to ionizing radiation following the Chernobyl nuclear disaster. Leukemia. (2020) 34:645–50. doi: 10.1038/s41375-019-0679-2, PMID: 31836850

[90] Gemitzi A. Are vegetation dynamics impacted from a nuclear disaster? The case of Chernobyl using remotely sensed NDVI and land cover data. Land. (2020) 9:433. doi: 10.3390/land9110433

[91] Gnatko OP Mizerna SD. On the consequences of the Chernobyl disaster: obstetrical aspects [До питання про наслідки Чорнобильської катастрофи: акушерські аспекти]. Neonatology, Surgery and Perinatal Medicine. (2016) 6:15–9. doi: 10.24061/2413-4260.VI.2.20.2016.2

[92] IAEA. Environmental impact assessment of the drawdown of the Chernobyl NPP cooling pond as a basis for its decommissioning and remediation. IAEA-TECDOC-1886 International Atomic Energy Agency (2019). Vienna, Austria: International Atomic Energy Agency

[93] Kandrychyn S. Regional differentiation in prevalence of mental retardation in Belarus after Chernobyl nuclear disaster. Psychiatry Psychother Clin Psychol. (2016) 7:165–75.

[94] Knowles SG. The meanings of a disaster: Chernobyl and its afterlives in Britain and France by Karena Kalmbach (review). Technol Cult. (2022) 63:906–7. doi: 10.1353/tech.2022.0140, PMID: 35848270

[95] Matsala M Bilous A Myroniuk V Holiaka D Schepaschenko D See L . The return of nature to the Chernobyl exclusion zone: increases in forest cover of 1.5 times since the 1986 disaster. Forests. (2021) 12:1024. doi: 10.3390/f12081024

[96] McCall C. Chernobyl disaster 30 years on: lessons not learned. Lancet. (2016) 387:1707–8. doi: 10.1016/S0140-6736(16)30304-X, PMID: 27116266

[97] Milovsky GA Ishmukhametova VT. Application of multispectral LANDSAT space imaging for the evaluation of radionuclide-contaminated sites in the Russian zone of the Chernobyl disaster on the example of Kaluga and Bryansk oblasts. Izvestiya. (2018) 54:1158–71. doi: 10.1134/S0001433818090244

[98] Naylor RL. Chernobyl, dark waters and the contingency of environmental disaster and scientific knowledge. Int Rev Environ Hist. (2022) 8:7–12. doi: 10.22459/IREH.08.02.2022.01

[99] Oe M Takebayashi Y Sato H Maeda M. Mental health consequences of the three Mile Island, Chernobyl, and Fukushima nuclear disasters: a scoping review. Int J Environ Res Public Health. (2021) 18:7478. doi: 10.3390/ijerph18147478, PMID: 34299933 PMC8304648

[100] Onopriyenko OP. Current angioneurology trends in medical care for stroke among the population, including those living in temporarily contaminated areas of Ukraine, and participants of elimination of Chernobyl accident consequences: modern view of the problem and own researches (to the 35th anniversary of the man-made disaster at the Chernobyl nuclear power plant). Int Neurol J. (2021) 17:31–41. doi: 10.22141/2224-0713.17.1.2021.226916

[101] Shrivastava SR Shrivastava PS Ramasamy J. After three decades of the Chernobyl nuclear disaster: where we are and what we have to focus upon. Ann Trop Med Public Health. (2017) 10:753–4. doi: 10.4103/1755-6783.196850

[102] Taha A Taha-Mehlitz S Nadyrov EA Zinovkin D Veyalkin I Levin L . Second primary cancer among patients with papillary thyroid carcinoma following the Chernobyl disaster. JAMA Netw Open. (2023) 6:e2329559. doi: 10.1001/jamanetworkopen.2023.29559, PMID: 37589974 PMC10436126

